# The prolonged health sequelae “of the COVID-19 pandemic” in sub-Saharan Africa: a systematic review and meta-analysis

**DOI:** 10.3389/fpubh.2025.1415427

**Published:** 2025-01-24

**Authors:** Melsew Setegn Alie, Getayeneh Antehunegn Tesema, Gossa Fetene Abebe, Desalegn Girma

**Affiliations:** 1 Department of Public Health, School of Public Health, College of Medicine and Health Science, Mizan-Tepi University, Mizan-Aman, Ethiopia; 2 Department of Epidemiology and Biostatistics, Institute of Public Health, College of Medicine and Health Sciences, University of Gondar, Gondar, Ethiopia; 3 School of Public Health and Preventive Medicine, Monash University, Melbourne, VIC, Australia; 4 Department of Midwifery, College of Medicine and Health Science, Mizan Tepi University, Mizan-Aman, Ethiopia

**Keywords:** long COVID-19, COVID sequalae, COVID-19, SARS CoV-2, sub-Saharan Africa

## Abstract

**Background:**

Survivors of coronavirus disease 2019 (COVID-19) often face persistent and significant challenges that affect their physical, mental, and financial wellbeing, which can significantly diminish their overall quality of life. The emergence of new symptoms or the persistence of existing ones after a severe acute respiratory syndrome coronavirus 2 (SARS-CoV-2) diagnosis has given rise to a complex clinical issue known as “long COVID-19” (LC). This situation has placed additional strain on global healthcare systems, underscoring the urgent need for ongoing clinical management of these patients. While numerous studies have been conducted on the long-term effects of COVID-19, our systematic review, and meta-analysis, is the first of its kind in sub-Saharan Africa, providing a comprehensive understanding of the situation in the region and highlighting the necessity for continuous clinical management.

**Objective:**

This study aimed to systematically synthesize evidence on the persistent sequelae of COVID-19 and their predictors in sub-Saharan Africa.

**Methods:**

A thorough search was conducted across multiple databases, including PubMed/MEDLINE, Web of Science, Google/Google Scholar, African online journals, and selected reference lists, from the inception of these databases until January 12, 2024. A meta-analysis of proportions was conducted using the random-effects restricted maximum-likelihood model. The association between various factors was also analyzed to determine the pooled factors that influence long COVID-19 outcomes.

**Results:**

Our comprehensive analysis of 16 research articles, involving a total of 18,104 participants revealed a pooled prevalence of COVID-19 sequelae at 42.1% (95% CI: 29.9–55.4). The long-term health sequelae identified in this review included persistent pulmonary sequelae (27.5%), sleep disturbance (22.5%), brain fog (27.4%), fatigue (17.4%), anxiety (22.3%), and chest pain (13.2%). We also found a significant association was observed between comorbidities and long COVID-19 sequelae [POR = 4.34 (95% CI: 1.28–14.72)], providing a comprehensive understanding of the factors influencing long COVID-19 outcomes.

**Conclusion:**

COVID-19 can have long-lasting effects on various organ systems, even after a person has recovered from the infection. These effects can include brain fog, pulmonary symptoms, sleep disturbances, anxiety, fatigue, and other neurological, psychiatric, respiratory, cardiovascular, and general symptoms. It is crucial for individuals who have recovered from COVID-19 to receive careful follow-up care to assess and reduce any potential organ damage and maintain their quality of life.

**Systematic review registration:**

Clinicaltrial.gov, identifier CRD42024501158.

## Introduction

Persistent coronavirus disease 2019 (COVID-19) is a condition where individuals continue to experience symptoms for weeks or even months after being infected with severe acute respiratory syndrome *coronavirus 2* (SARS-CoV-2) (1). COVID-19 is the term coined first during the spring season of 2020 through a social media movement, where people with suspected or confirmed COVID-19 infections were not recovering even weeks after the onset of their symptoms (2, 3). It is important to note that long COVID-19 can result irrespective of the severity of the initial infection (4, 5). While the mechanisms underlying this condition are still largely unknown, it is early to label all of its indicators as a postviral illness (3, 6). Although evidence describing this condition is scarce, emerging research is shedding light on the long-term health impairment and organ damage that can result from COVID-19 infection (7–10). Unfortunately, COVID-19-infected patients face challenges in accessing adequate recognition, support, medical assessment, and treatment for their condition—especially during the first wave of the pandemic when testing was not readily available to those who did not have laboratory-tested evidence and to those who were not hospitalized during the initial phase of the infection (11, 12).

COVID-19 survivors frequently encounter long-lasting and consequential medical, psychological, and financial difficulties that can further reduce their quality of life (13). Despite the ongoing vaccination campaigns (14), the health implications of COVID-19 continue to be a significant concern, as the long-term effects on various bodily systems are not yet comprehended completely. As patients exhibit a broad spectrum of clinical indicators and degrees of severity, it is critical to gain a more comprehensive understanding of the emergence of new symptoms or the persistence of existing ones regarding COVID-19 infection (15).

According to a December 22, 2023 report, Africa had reported more than 9.5 million confirmed cases of COVID-19, accounting for only 1% of the global confirmed cases exceeding 772 million (16). However, limited research has been conducted on the impact of long COVID-19 in Africa, with only a few studies exploring the prevalence and burden of long COVID-19 on the continent. The prevalence of COVID-19 has been found to vary across African countries and even within them. Notably, a study conducted in South Africa reported the highest prevalence (66.7%) compared to other studies conducted worldwide (17). Previously, there was no pooled study on the long COVID-19 sequelae and its determinants in sub-Saharan Africa. This study aimed to close the gap of the lack of a pooled prevalence by doing a systematic review and meta-analysis.

## Methods

### Research questions

To conduct a systematic review of the pooled prevalence of long COVID-19 signs and symptoms in sub-Saharan Africa, the research question was structured using the PICO(S) format. The participants (P) were clinically diagnosed or laboratory-confirmed individuals to have COVID-19 infection. Long COVID-19 is the presence of signs and/or symptoms that cannot be attributed to any other medical condition. The intervention (I) and comparison (C) of this systematic review and meta-analysis were none, while the outcome (O) was the incidence of signs and symptoms of long COVID-19 in sub-Saharan African countries. The study design (S) was all observational studies. Our research questions for this study were as follows: The first one was what is the long COVID-19 sequelae in sub-Saharan Africa? The second one was what are the influencing factors of long COVID-19 sequelae in sub-Saharan Africa?

### Case definition

Long COVID-19: Post-COVID-19 condition refers to a set of symptoms that occur in individuals who have a history of probable or confirmed SARS-CoV-2 infection (18). In this study, long COVID-19 refers to a significant presence of persistent symptoms lasting more than 4 weeks after the SARS-CoV-2 infection.

### Protocol and registration

This systematic review and meta-analysis was registered in the International Prospective Register of Systematic Reviews (PROSPERO), and the identification number of this systematic review and meta-analysis is CRD42024501158.

### Data sources and search strategy

This systematic review and meta-analyses were conducted by the Preferred Reporting Items for Systematic Reviews and Meta-Analyses (PRISMA) reporting guideline (19). We performed an extensive literature search using various electronic bibliographic databases, such as PubMed/MEDLINE, Web of Science, Google/Google Scholar, and African online journals. The main objective of this study was to obtain accurate and reliable results; therefore, we employed a combination of keywords and a relevant thesaurus focused on long COVID-19 and its associated risk factors. To identify publications discussing long COVID-19 in the general population, we used the following search terms: “COVID-19 OR COVID OR SARSCOV-2 OR coronavirus OR long-COVID-19 OR post-COVID” AND “PASC OR haulers OR lingering OR postacute OR persistent OR convalescent OR convalescence OR sequelae OR postviral” AND “testing and smell change” AND “post-COVID symptom and cardiopulmonary or pulmonary symptom post-COVID-19” AND “psychiatric and mental symptom post-COVID-19” AND “physical or cognitive symptom post-COVID-19” AND “Adult or healthcare provider or general population or high-risk population or cancer patients or chronic illness patient or HIV positive individuals or immune-compromised individuals or pediatric OR kids OR young OR infant OR children OR adolescents.” We focused on all observational studies conducted in sub-Saharan Africa. Two authors (GF and DG) independently screened titles, abstracts, and full texts of articles. In the cases of disagreement regarding the inclusion of a full-text article, all the authors participated in discussions to reach a consensus.

### Inclusion criteria

The study conducted in sub-Saharan Africa among adults older than 18 years with an observational study design from January 1, 2020 to January 12, 2024 and published in English, which measured long COVID-19 sequelae, were included in this systematic review and meta-analysis.

### Exclusion criteria

Our systematic review and meta-analysis excluded qualitative studies, communications letters, reviews, commentaries, letters to the editors, studies where the raw data cannot be analyzed, and protocols conducted in sub-Saharan Africa.

### Study data management

After conducting a comprehensive search and gathering multiple articles, we eliminated duplicate files. This screening process involved two stages: initially assessing the titles and abstracts and conducting a full-text screening. To ensure accuracy, two independent reviewers (MS and DG) utilized the EndNote software to evaluate the potential relevance of each article for further review. The assessment was based on a predefined set of inclusion and exclusion criteria. In the cases where discrepancies existed between the reviewers’ assessments, they were resolved through discussion and inputs from a third reviewer. For auditing, electronic records were maintained for both the included and the excluded studies, with clear explanations if there were any exclusions.

### Risk of bias evaluation and quality assessment

To assess the risk of bias in the study, a quality assessment checklist for prevalence studies was employed. This checklist, developed by Hoy and colleagues, consists of nine items that are crucial in evaluating the quality of a study (20). These items include the target population, sampling frame, sampling method, response rate, data collection procedures, study case definition, study instruments, and the numerator and denominator parameters. Each item contributes to a total score of 9. Based on the scores obtained, the studies were categorized as having a high-risk (0–3), moderate-risk (4–6), or low-risk (7–9) bias. Each study underwent an independent evaluation, and the majority of them demonstrated a low risk of bias. To ensure the reliability of the results, studies with a high risk of bias were excluded from the final analysis.

### Data extraction

The data extraction process utilized a Microsoft Excel template 2013. The form underwent iterative testing and revision as necessary. Two authors (MS and DG) independently extracted the data using a standardized form. The extracted descriptive variables encompassed various aspects such as country, study design, study period, data collection method, sample size, age, sex, outcomes, factors associated with long COVID-19, and the terminology employed to describe the long-term effects of COVID-19.

### Sensitivity analyses

A thorough sensitivity analysis was conducted to assess how individual studies affected the overall prevalence estimation. Each study was methodically removed, and the impact on the estimate was carefully examined. The exclusion of any single study did not significantly affect the pooled prevalence estimate. Furthermore, none of the studies fell outside the confidence interval’s lower and upper boundaries.

### Data synthesis and analysis

Data entry was conducted using Microsoft Excel 2013 and subsequently exported to R software version 4.2^2^ for the analysis. The meta-package was employed to analyze the data. Heterogeneity was assessed using the *I*^2^ statistic (21), with thresholds for low, medium, and high heterogeneity set at 25, 50, and 75%, respectively (22).

Subgroup analyses were performed based on population characteristics and sample sizes. To identify potential sources of heterogeneity, meta-regression analysis was carried out, considering the sample size and publication years. Sensitivity analyses were conducted by excluding individual studies to evaluate the impact of each study on the overall pooled prevalence of long COVID-19 sequelae in sub-Saharan Africa. A funnel plot was visually inspected to assess publication bias, followed by Egger’s test to determine any significant publication bias. The results were presented using forest plots and tables. A random-effects model was utilized to estimate the pooled prevalence of long COVID-19 health sequelae, and its determinants were identified and presented with an odds ratio with a 95% confidence interval (CI).

## Results

### Study identification and selection

**Figure 1:**
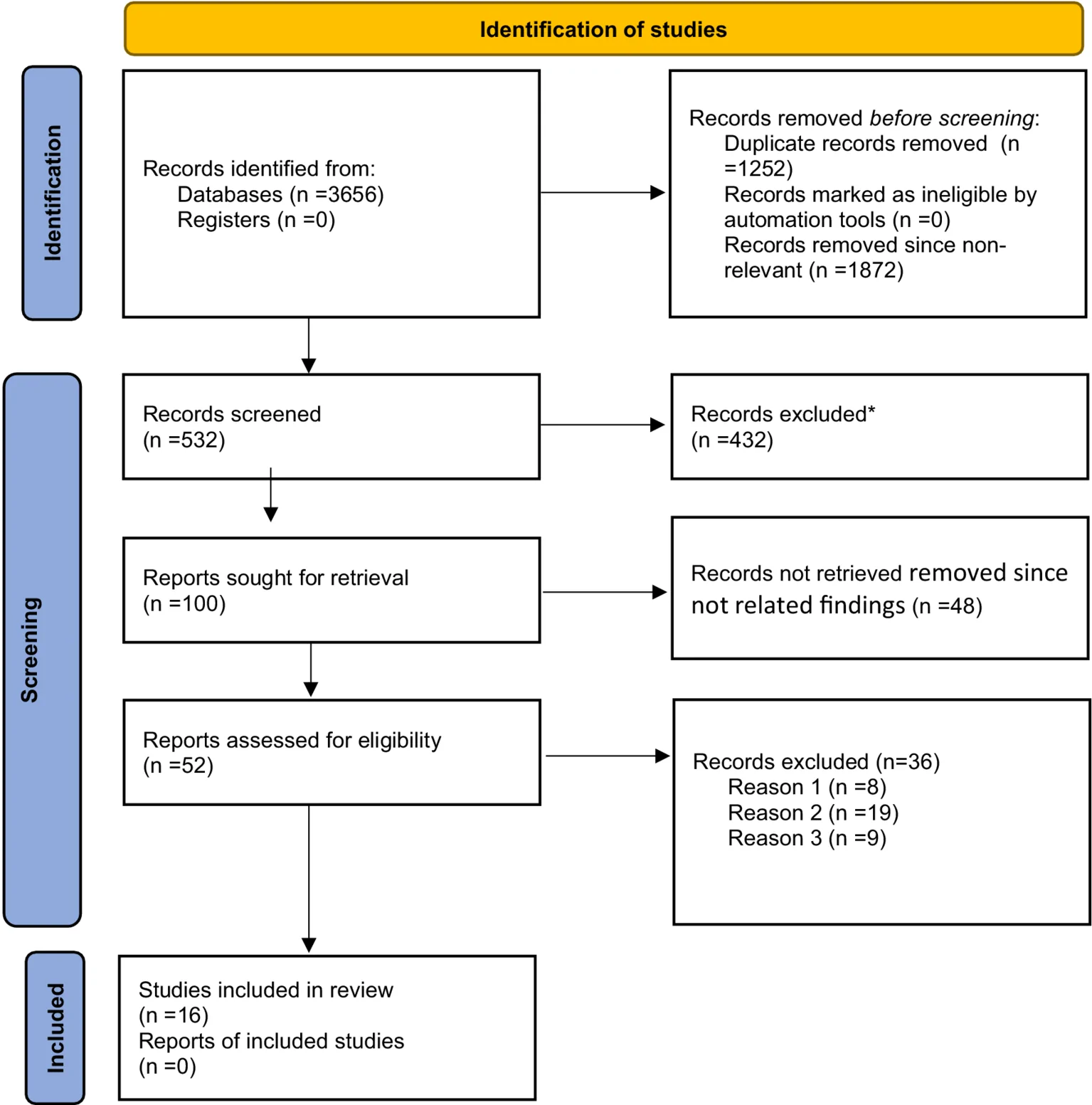
The PRISMA flow chart shows study selection for systematic review and meta-analysis of long COVID-19 sequelae in sub-Saharan Africa. *Excluded by title and abstract. Reason 1: Not directly related finding, published in non-English languages, and editorial letter. Reason 2: Qualitative finding, review protocol, unclear outcome measurement. Reason 3: Protocol, inaccessibility of full document, review paper.

**Figure 2:**
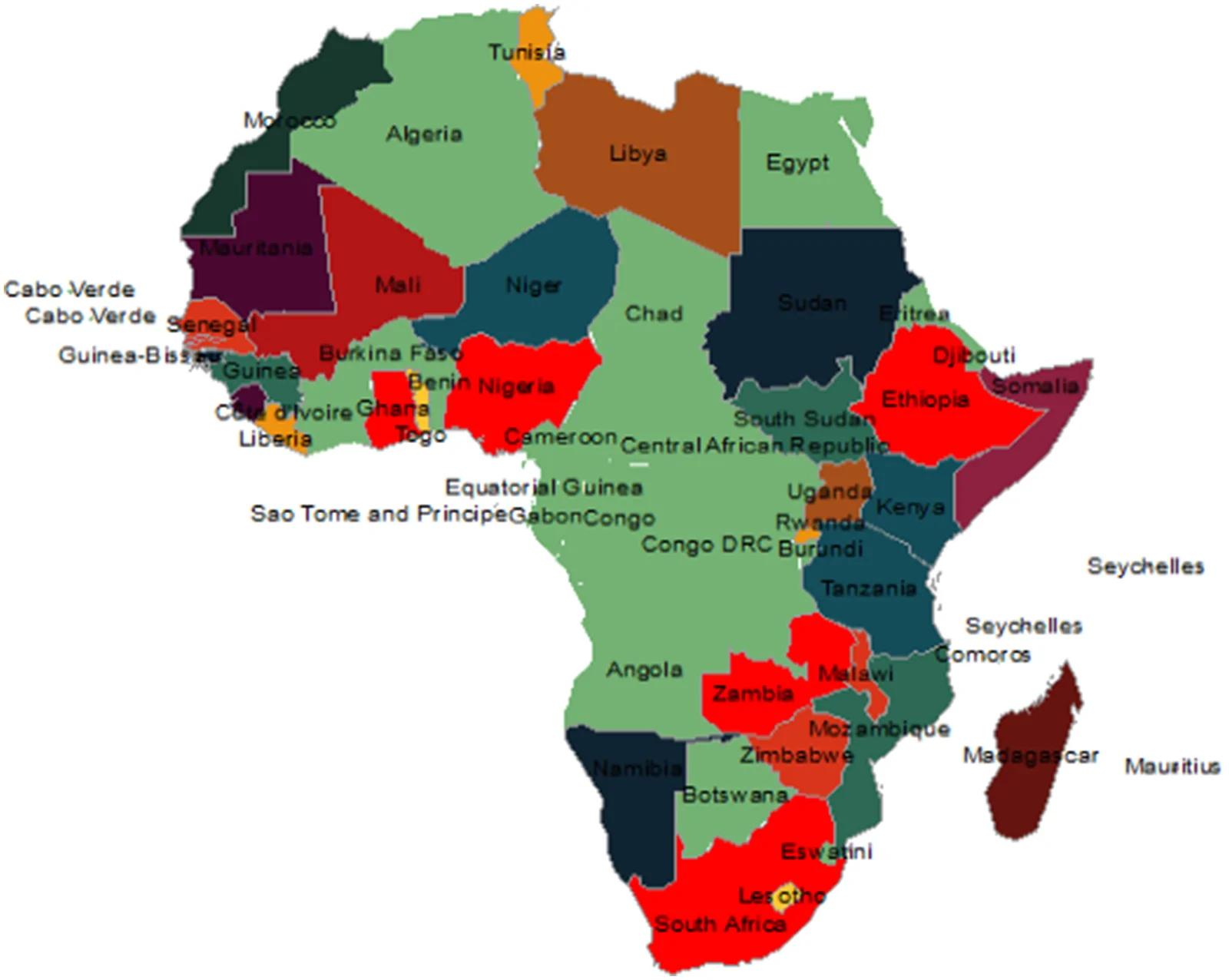
The country that experienced the long COVID-19 sequelae in sub-Saharan Africa (red). Source: Shapefile of https://www.africageoportal.com/.

Initially, a total of 3,656 articles were identified through electronic search databases. Using the endnote application, 1,252 duplicate records were removed, discarding 2,404 records for further assessment. Of these, 1,872 articles were deemed irrelevant and were removed from the library. The remaining 532 articles underwent additional screening based on their titles and abstracts. From this screening, 432 articles were excluded due to unrelated findings, resulting in 100 articles for full-text screening. Of these, 48 articles were excluded due to unrelated findings, qualitative study design, and non-English publication, discarding only 52 articles for further assessment. In the last step, 36 articles were excluded from the analysis due to incomplete reporting of the desired outcome, qualitative study design, a study protocol, qualitative study, study out of sub-Saharan Africa, and inaccessibility of the full text. Finally, only 16 articles were included for systematic review and meta-analysis (Figure 1). The identified studies are geographically from Ethiopia, Ghana, South Africa, and Zambia. The geographic location of the survey of COVID-19 sequelae is presented in red color in Figure 2. In the map, the countries colored in red show the area of long COVID-19 sequelae. Those countries have identified the evidence of long COVID-19 sequelae, while other sub-Saharan African countries may or may not assess the COVID-19 sequelae (Figure 2).

### Characteristics of the studies

This analysis comprised a total of 16 studies, which were conducted in five countries; among which, 50% (8 studies) were carried out in South Africa, and 3 studies were conducted in Ethiopia. The sample sizes in these studies varied, ranging from 30 (23) to 2,840 (24) participants, resulting in a total sample size of 18,104 participants. The participants included in these studies were from diverse backgrounds, including the general population and healthcare providers. The earliest survey was conducted in 2021, while the most recent one was conducted in 2023 (Table 1).

**Table 1:** Descriptive presentation of the study included in systematic review and meta-analysis.

References	Country	Sample size	Population	Study period
Elias et al. (69)	Ethiopia	340	General population	June 12, 2020 and November 1, 2021
Engida et al. (70)	Ethiopia	573	General population	June 1 to 20, 2020
Seyoum et al. (71)	Ethiopia	405	General population	January 2021 and January 2022
Nuamah et al. (72)	Gahanna	253	General population	April 1, 2020 to March 31, 2021
Ogoina et al. (23)	Nigeria	30	General population	April and December 2020
Osikomaiya et al. (73)	Nigeria	274	General population	April and June 2020
Jassat et al. (24)	South Africa	2,366	Hospitalized patients	Not reported
Jassat et al. (24)	South Africa	2,840	Hospitalized patients	Not reported
Jassat et al. (24)	South Africa	2,626	Hospitalized patients	Not reported
Kinge et al. (74)	South Africa	62	Health workers	15 February to 15 April 2021
Dryden et al. (17)	South Africa	2,410	General population	Dec 1, 2020 and August 23, 2021
Dryden et al. (17)	South Africa	1,873	General population	Dec 1, 2020, and August 23, 2021
Dryden et al. (17)	South Africa	2,413	General population	December 2020 and August 2021
Kruger et al. (75)	South Africa	99	Long COVID-19 patients	Not reported
Malambo et al. (76)	Zambia	1,238	General population	August 2020 to January 2023
Zulu et al. (77)	Zambia	302	General population	March 18, 2020 to March 30, 2021

### Risk of bias evaluation

The Newcastle–Ottawa Scale (NOS) was used to evaluate the quality and potential bias of the studies. This assessment system considers three key parameters: selection, comparability, and outcome. A maximum of 9 points can be assigned, with one author conducting the assessment and another author independently reviewing it to ensure accuracy. The total score determines the level of bias risk, which is categorized as high (0–3), moderate (4–6 points), or low (7–9 points) (25) (Supplementary Table S1). Based on the risk of bias evaluation, the studies included in this systematic review and meta-analysis were low.

### Meta-analysis of long COVID-19 sequelae

**Figure 3:**
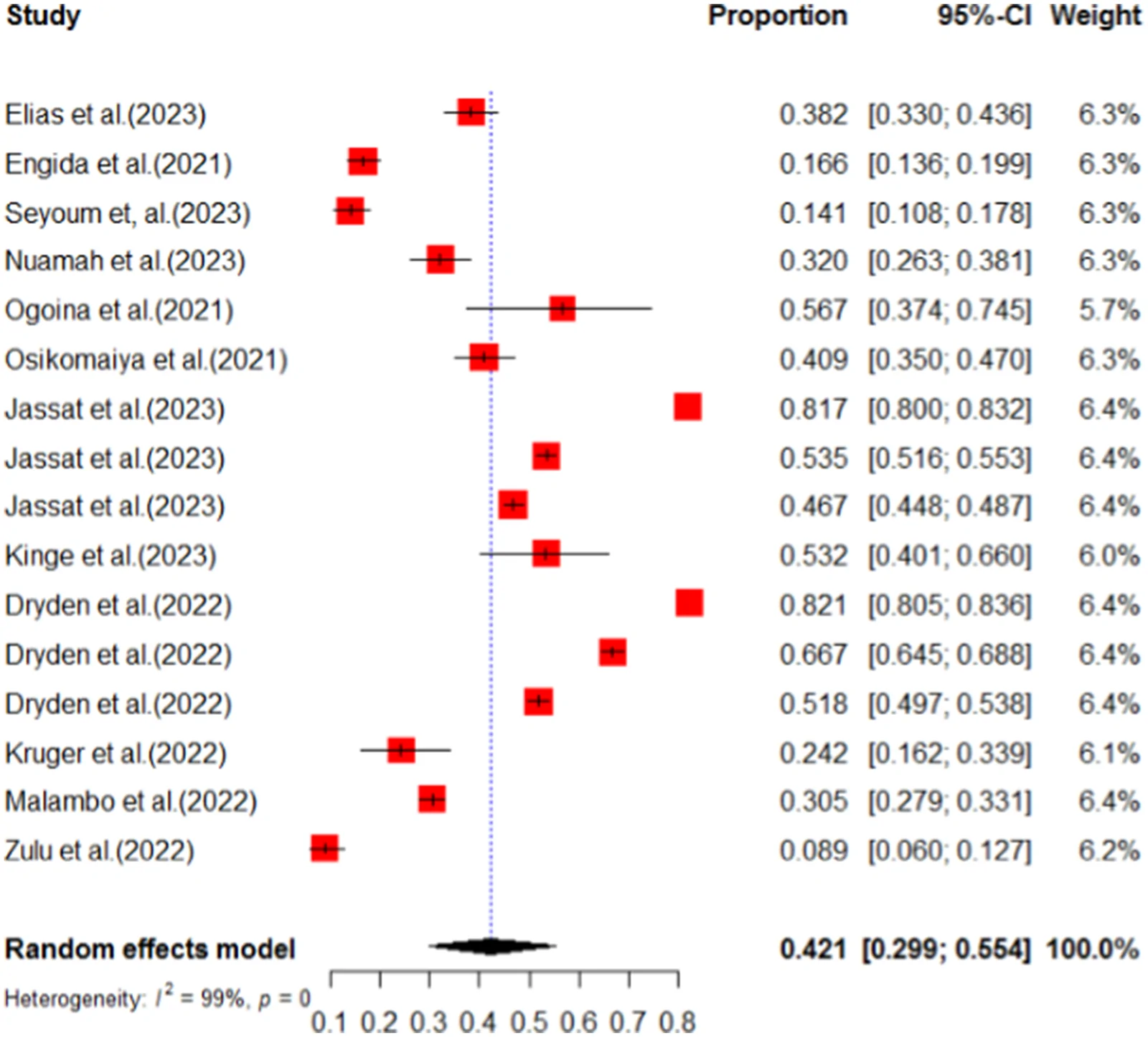
Occurrence of persistent symptoms of COVID-19 in sub-Saharan Africa.

The study conducted a systematic review and meta-analysis to investigate the symptomatology of long COVID-19 sequelae in sub-Saharan Africa. A total of 16 publications were analyzed, representing research from various regions, including South Africa, Ethiopia, Nigeria, Ghana, and Zambia. The analysis focused on persistent COVID-19 sequelae across different categories, such as neurological, psychiatric, respiratory, cardiovascular, and general symptoms. The pooled data from 16 studies indicated that the prevalence of persistent COVID-19 symptoms in sub-Saharan Africa was 42.1% (95% CI: 29.9–55.4) (Figure 3). Furthermore, a sensitivity analysis was conducted to evaluate the main outcomes and each specific symptom associated with long COVID-19 sequelae. This analysis involved systematically removing one study at a time to assess the impact of each study on the overall effect estimate. The results showed that the removal of any single study did not significantly alter the pooled prevalence. Additionally, all studies remained within the established lower and upper limits of the confidence interval.

### Subgroup analysis

**Figure 4:**
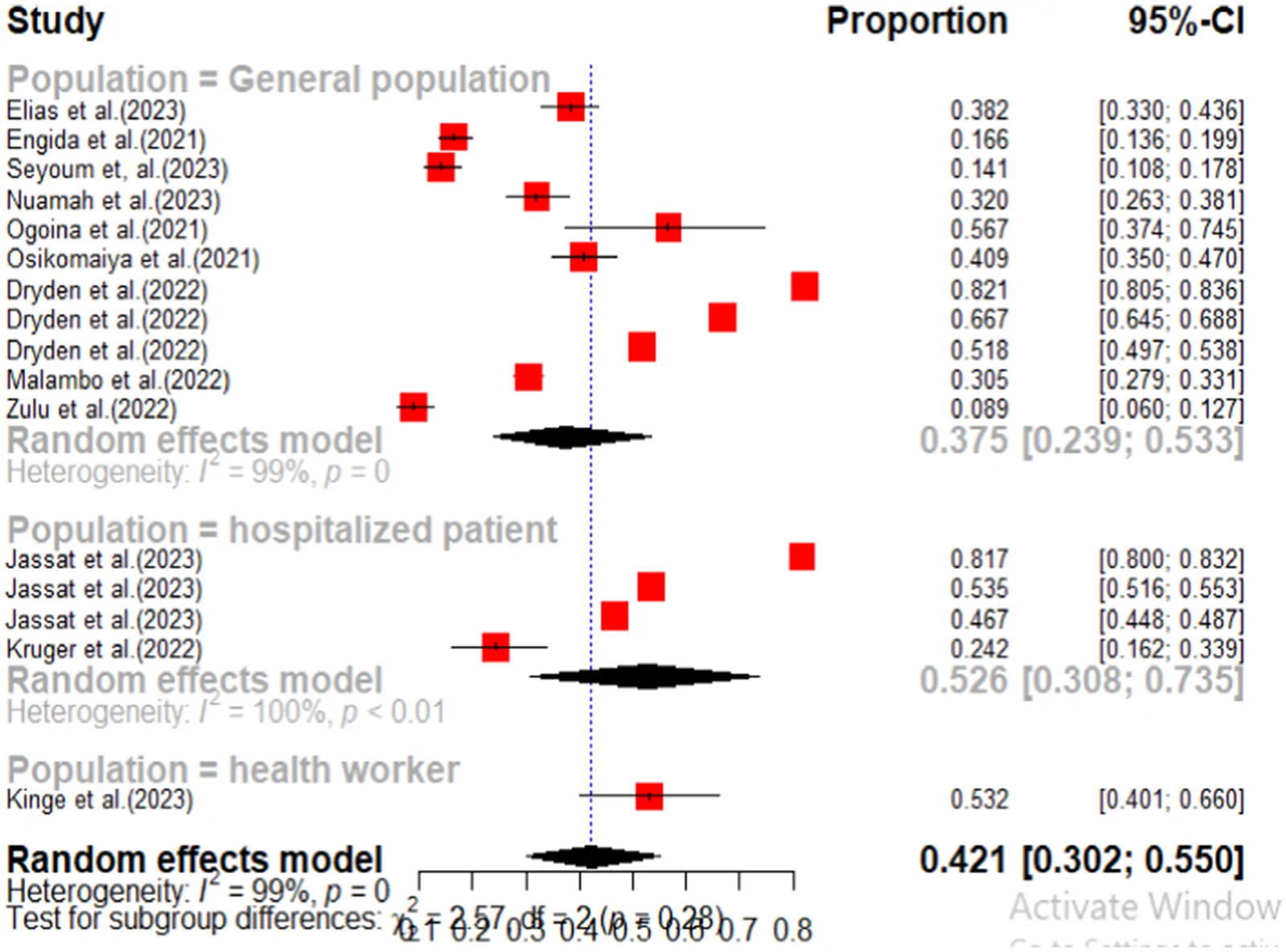
Subgroup analysis of COVID-19 sequelae based on population in sub-Saharan Africa.

**Figure 5:**
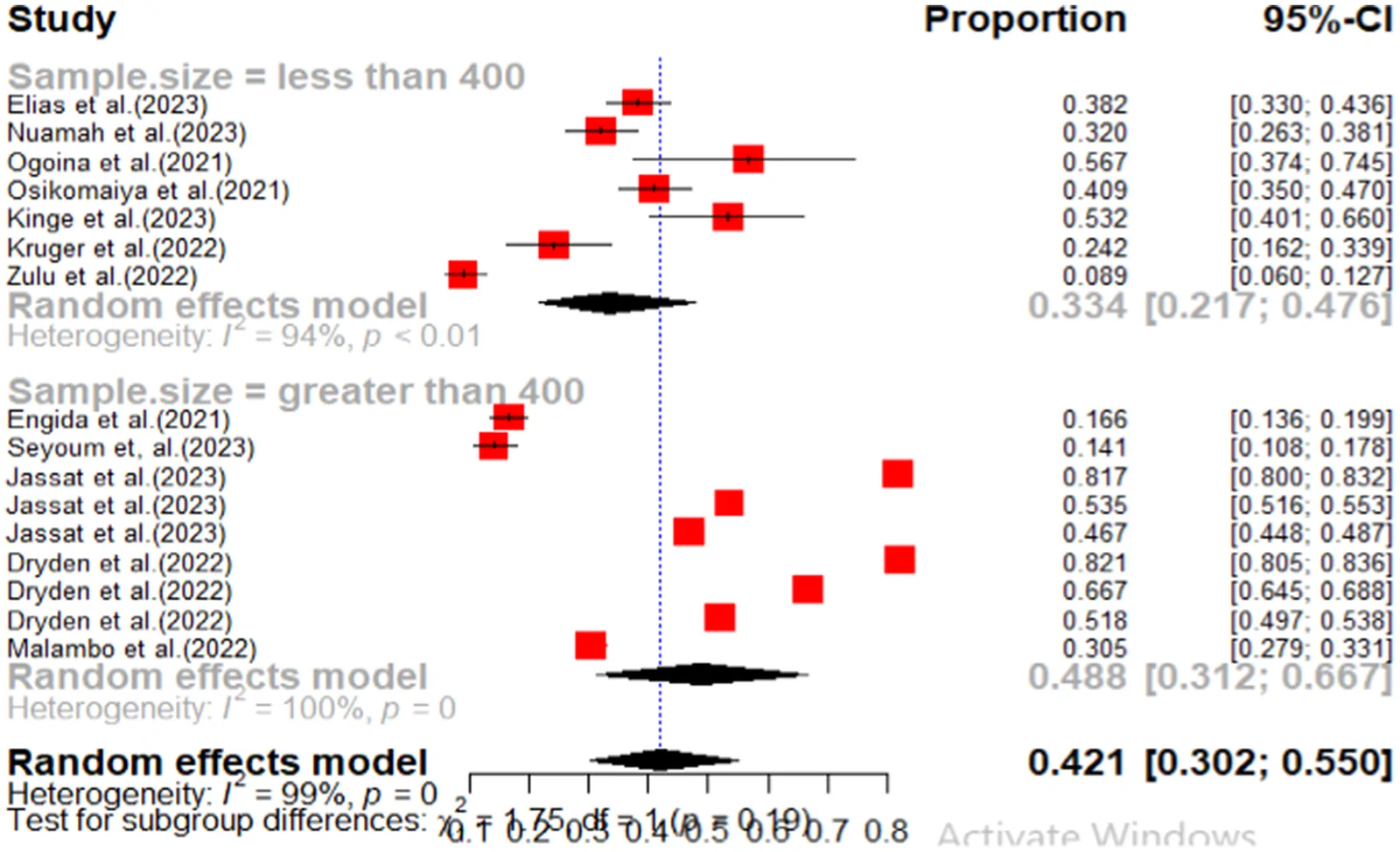
Subgroup analysis of COVID-19 sequelae based on sample size in sub-Saharan Africa.

According to this systematic review and meta-analysis, subgroup analyses were performed based on population type and sample size. The findings indicated that hospitalized individuals experienced the highest prevalence of COVID-19 sequelae, with a pooled prevalence of COVID-19 sequelae were 52.6% (95% CI: 30.2–55.0), followed by the general population at 37.5% (95% CI: 23.9–53.3) (Figure 4). Additionally, the analysis based on sample size revealed that studies with a sample size greater than 400 reported a higher prevalence of COVID-19 sequelae at 48.8% (95% CI: 31.2–66.7), compared to those with a sample size of less than 400 (Figure 5).

### Subanalysis by country

**Figure 6:**
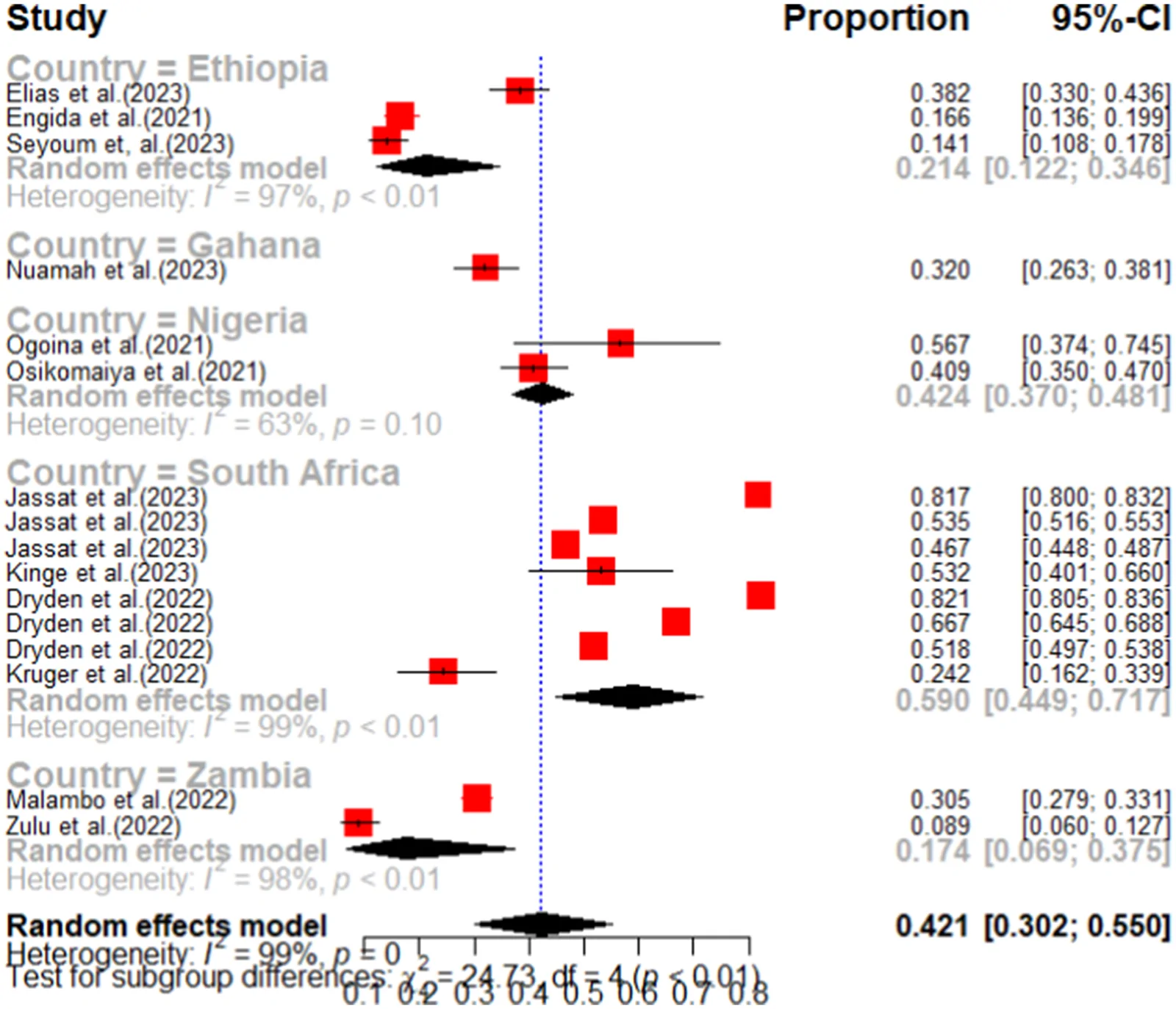
The forest plot of subgroup analysis based on the country.

Based on a subanalysis by country, South Africa exhibited the highest pooled prevalence of COVID-19 sequelae at 59.0% (95% CI: 44.9–71.7). Following closely, Nigeria recorded the second highest prevalence, with a rate of 42.4% (95% CI: 37.0–48.1). A detailed sub-country analysis is illustrated in Figure 6.

### Meta-bias and publication bias

The funnel plot analysis examining the impact of smaller studies on the overall COVID-19 sequelae in sub-Saharan Africa revealed a small study effect. The funnel plot exhibited asymmetry, prompting further investigation through Egger’s test. For a visual representation of the funnel plot, please refer to Supplementary Figure S1. The results of Egger’s test indicated a non-significant outcome, with a *p*-value of 0.1480 and B0 = −8.7670 (SE = 5.7254), leading to the conclusion that further analysis was unnecessary. Additionally, the meta-bias analysis found that neither publication year nor sample size were statistically significant sources of heterogeneity. The results of the meta-regression analysis, as shown in Table 2, confirmed that both sample size and publication year did not significantly contribute to heterogeneity.

**Table 2:** Meta-regression analysis of factors affecting between-study heterogeneity long COVID-19 sequelae in sub-Saharan Africa.

Heterogeneity source	Coefficients	Standard error	*p*-value
Sample size	0.0006	0.0002	0.1800
Publication year	0.1867	0.3518	0.5958

### General sequelae of long COVID-19

In this category, we have synthesized the combined prevalence of various symptoms associated with long COVID-19 in sub-Saharan Africa. Among these symptoms, fatigue exhibited the highest pooled prevalence at 17.4% (95% CI: 7.1–36.7), accompanied by a significant level of heterogeneity (*I*^2^) at 99.0%. Myalgia was the second most common symptom identified in this systematic review and meta-analysis, with a pooled prevalence of 3.9% (95% CI: 1.1–12.3). A detailed overview of the general sequelae of long COVID-19 can be found in Table 3.

**Table 3:** The general sequelae of COVID-19 in sub-Saharan Africa.

COVID-19 sequelae	Studies	Proportion (95% CI)
Fatigue	Elias et al. (2023)	0.259 (0.213–0.309)
	Ogoina et al. (2021)	0.267 (0.123–0.459)
	Osikomaiya et al. (2021)	0.128 (0.091–0.173)
	Jassat et al. (2023)	0.028 (0.022–0.035)
	Jassat et al. (2023)	0.014 (0.010–0.019)
	Jassat et al. (2023)	0.012 (0.008–0.017)
	Kinge et al. (2023)	0.419 (0.295–0.552)
	Dryden et al. (2022)	0.509 (0.489–0.529)
	Dryden et al. (2022)	0.503 (0.480–0.526)
	Dryden et al. (2022)	0.508 (0.488–0.528)
	Kruger et al. (2022)	0.737 (0.639–0.821)
	Malambo et al. (2022)	0.385 (0.358–0.413)
	Zulu et al. (2022)	0.013 (0.004–0.034)
Random effect: *I*^2^ = 99%, *p* = 0.01		**0.174 (0.071–0.367)**
Myalgia	Ogoina et al. (2021)	0.067 (0.008–0.221)
	Osikomaiya et al. (2021)	0.088 (0.057–0.128)
	Jassat et al. (2023)	0.009 (0.006–0.014)
	Jassat et al. (2023)	0.007 (0.004–0.010)
	Jassat et al. (2023)	0.003 (0.002–0.006)
	Kinge et al. (2023)	0.210 (0.117–0.33)
	Kruger et al. (2022)	0.0.485 (0.383–0.587)
	Malambo et al. (2022)	0.141 (0.122–0.162)
	Zulu et al. (2022)	0.007 (0.001–0.024)
Random effect: *I*^2^ = 99%, *p* < 0.01		**0.039 (0.011–0.123)**
Loss of appetites	Elias et al. (2023)	0.053 (0.032–0.082)
	Osikomaiya et al. (2021)	0.088 (0.057–0.128)
	Dryden et al. (2022)	0.005 (0.003–0.009)
	Dryden et al. (2022)	0.011 (0.007–0.016)
	Dryden et al. (2022)	0.005 (0.003–0.009)
	Zulu et al. (2022)	0.013 (0.004–0.034)
Random effect: *I*^2^ = 96%, *p* < 0.01		**0.016 (0.006–0.039)**

CI, confidence interval. The bold values shows the “pooled result of the each sequalae.”

### Neurologic sequelae of long COVID-19

Among the neurological sequelae associated with COVID-19, brain fog exhibited the highest prevalence among persistent COVID-19 symptoms, with a pooled occurrence rate of 27.4% (95% CI: 7.2–64.8). This indicates that, out of 100 individuals, approximately 27 experienced ongoing symptoms of brain fog. The second most common neurological sequelae were loss of taste and loss of smell. Based on an analysis of six primary studies, the pooled occurrence of persistent loss of taste was found to be 7.5% (95% CI: 3.6–15.1), while the pooled occurrence of loss of smell was also 7.5% (95% CI: 3.7–14.6). As detailed in Table 3, the neurological sequelae of COVID-19 were consistently reported of exceeding 5%. The least prevalent persistent symptom identified was lack of concentration, which was reported as 5.4% (95% CI: 1.5–17.8), as shown in Table 4.

**Table 4:** Shows neurological sequelae of COVID-19 in sub-Saharan Africa.

COVID-19 sequelae	Studies	Proportion (95% CI)
Loss of taste	Elias et al. (2023)	0.024 (0.010–0.046)
	Nuamah et al. (2023)	0.198 (0.150–0.252)
	Dryden et al. (2022)	0.067 (0.058–0.078)
	Dryden et al. (2022)	0.027 (0.020–0.036)
	Dryden et al. (2022)	0.067 (0.057–0.078)
	Kruger et al. (2022)	0.253 (0.171–0.350)
Random effect: *I*^2^ = 97%, *p* < 0.01		**0.075 (0.036–0.151)**
Lack of concentration	Ogoina et al. (2021)	0.100 (0.021–0.265)
	Osikomaiya et al. (2021)	0.051 (0.028–0.084)
	Jassat et al. (2023)	0.008 (0.005–0.012)
	Jassat et al. (2023)	0.004 (0.002–0.007)
	Jassat et al. (2023)	0.004 (0.002–0.007)
	Dryden et al. (2022)	0.132 (0.119–0.146)
	Dryden et al. (2022)	0.175 (0.158–0.193)
	Dryden et al. (2022)	0.132 (0.119–0.146)
	Kruger et al. (2022)	0.707 (0.607–0.794)
Random effect: *I*^2^ = 99%, *p* < 0.01		**0.054 (0.015–0.178)**
Loss of Smell	Elias et al. (2023)	0.053 (0.032–0.082)
	Nuamah et al. (2023)	0.198 (0.150–0.252)
	Dryden et al. (2022)	0.051 (0.043–0.061)
	Dryden et al. (2022)	0.023 (0.017–0.031)
	Dryden et al. (2022)	0.051 (0.043–0.0061)
	Kruger et al. (2022)	0.253 (0.171–0.350)
Random effect: *I*^2^ = 97%, *p* < 0.01		**0.075 (0.037–0.146)**
Headache	Elias et al. (2023)	0.112 (0.080–0.150)
	Nuamah et al. (2023)	0.387 (0.327–0.450)
	Ogoina et al. (2021)	0.033 (0.001–0.172)
	Osikomaiya et al. (2021)	0.128 (0.091–0.173)
	Jassat et al. (2023)	0.009 (0.006–0.014)
	Jassat et al. (2023)	0.015 (0.011–0.021)
	Jassat et al. (2023)	0.004 (0.002–0.007)
	Dryden et al. (2022)	0.168 (0.154–0.184)
	Dryden et al. (2022)	0.138 (0.122–0.154)
	Dryden et al. (2022)	0.168 (0.154–0.184)
	Malambo et al. (2022)	0.148 (0.128–0.169)
	Zulu et al. (2022)	0.023 (0.009–0.047)
Random effect: *I*^2^ = 98%, *p* < 0.01		**0.061 (0.027–0.133)**
Blurring of vision	Osikomaiya et al. (2021)	0.022 (0.008–0.047)
	Dryden et al. (2022)	0.093 (0.082–0.105)
	Dryden et al. (2022)	0.101 (0.088–0.116)
	Dryden et al. (2022)	0.093 (0.082–0.105)
Random effect: *I*^2^ = 80%, *p* < 0.01		**0.073 (0.043–0.120)**
Brain fog	Kinge et al. (2023)	0.210 (0.117–0.332)
	Kruger et al. (2022)	0.707 (0.607–0.794)
	Malambo et al. (2022)	0.080 (0.065–0.096)
Random effect: *I*^2^ = 99%, *p* < 0.01		**0.274 (0.072–0.648)**

The bold values shows the “pooled result of the each sequalae.”

### Psychiatric sequelae of long COVID-19

**Figure 7:**
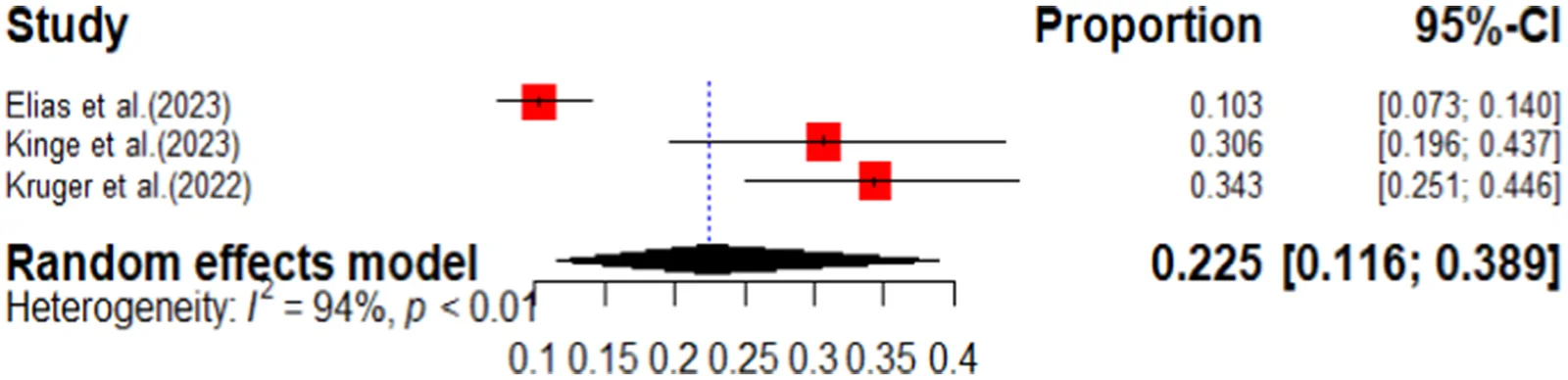
Forest plot of pooled prevalence of sleep disturbance as long COVID-19 sequelae in sub-Saharan Africa.

**Figure 8:**
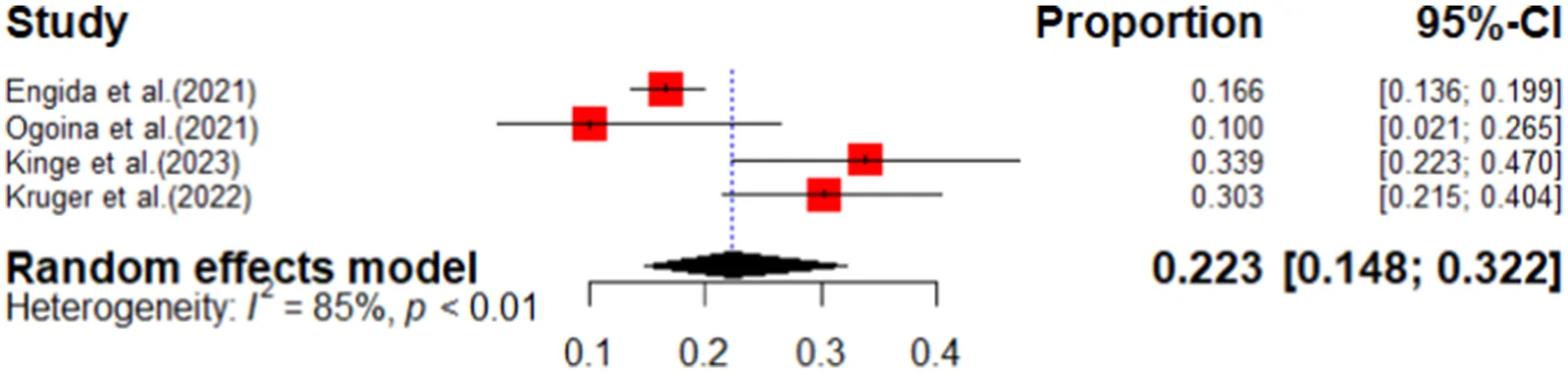
The forest plot of pooled prevalence of anxiety among long COVID-19 patients in sub-Saharan Africa.

This systematic review and meta-analysis investigated the psychiatric consequences of long COVID-19, drawing on data from ten different studies. The research focused on two primary psychiatric manifestations associated with long COVID-19. The most common psychiatric consequence identified was sleep disturbance, with a pooled prevalence of 22.5% (95% CI: 11.6–38.9). The studies exhibited a high level of heterogeneity, with an I^2^ statistic of 98% (Figure 7). The second most prevalent psychiatric issue among long COVID-19 patients was anxiety, which had a pooled prevalence of 22.3% (95% CI: 14.8–32.2). This finding also demonstrated significant heterogeneity (*I*^2^ = 85%) and a highly significant *p*-value (*p* < 0.01) (Figure 8).

### Respiratory sequelae of long COVID-19

**Figure 9:**
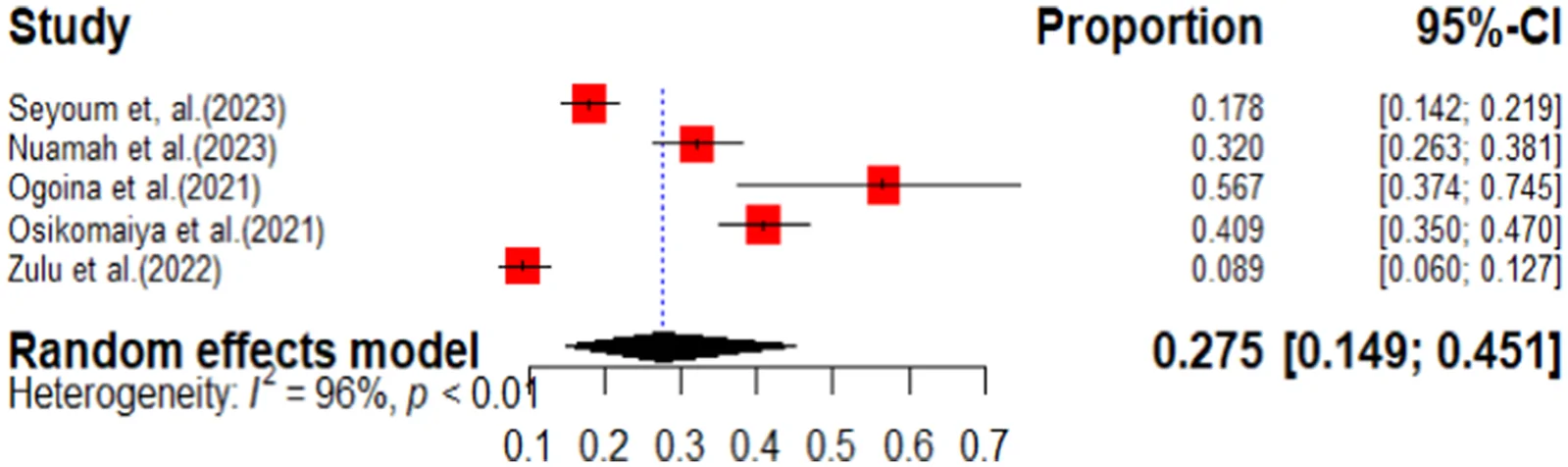
The forest plot of general pulmonary sequelae of long COVID-19 in sub-Saharan Africa.

**Figure 10:**
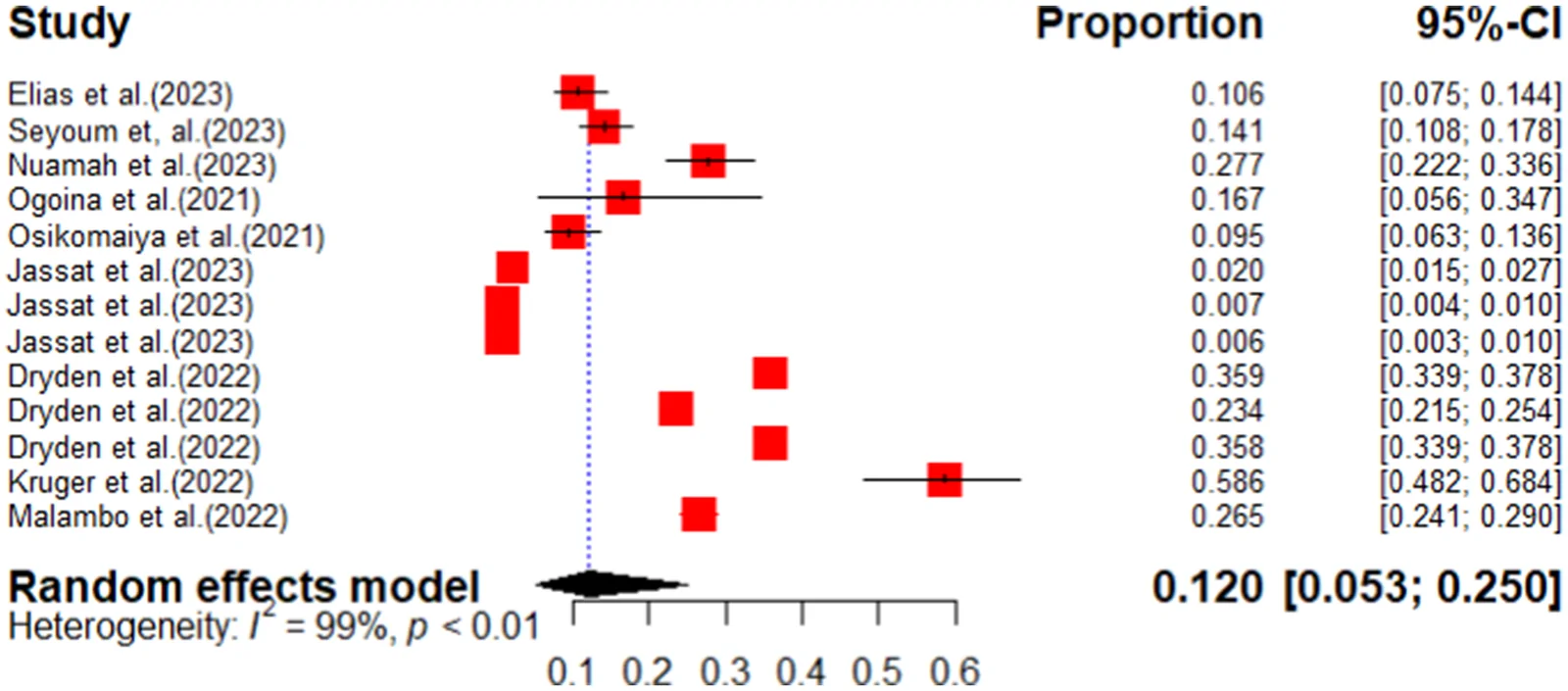
The forest plot of shortness of breath of COVID-19 sequelae in sub-Saharan Africa.

**Figure 11:**
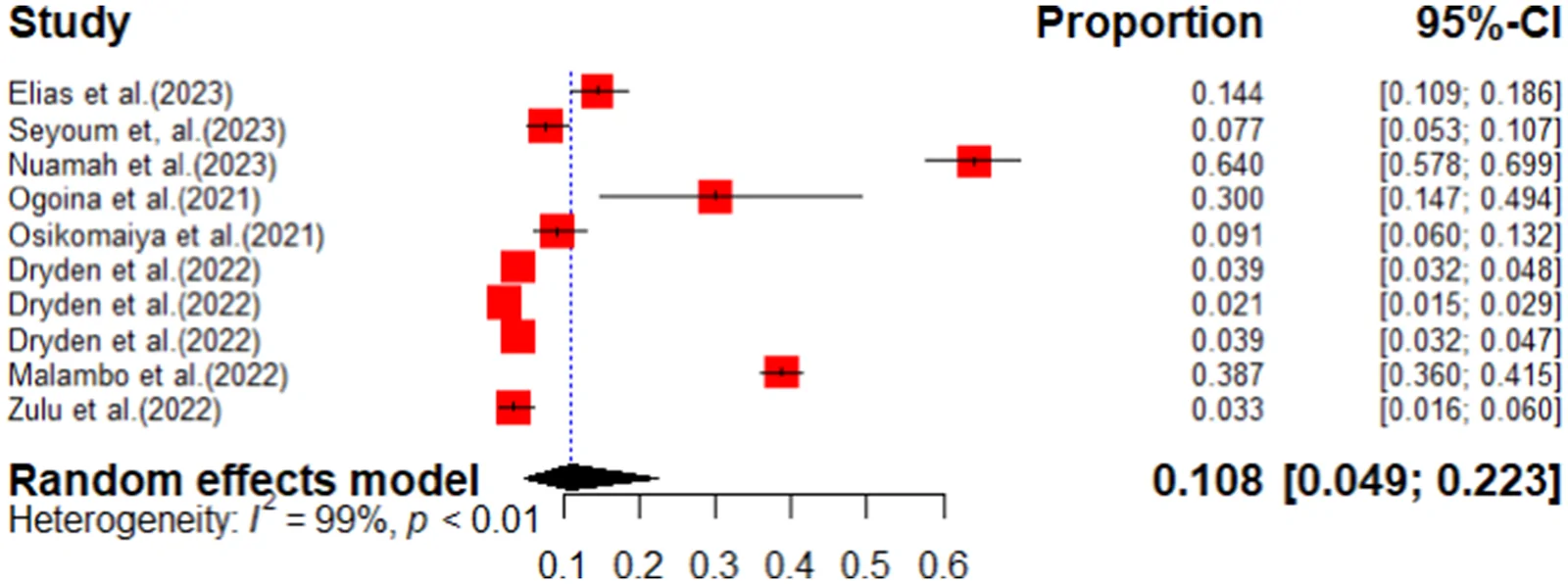
The forest plot of cough health sequelae of long COVID-19 in sub-Saharan Africa.

This review identified three primary respiratory sequelae: cough, shortness of breath, and general pulmonary complications. Among these, general pulmonary complications had the highest prevalence, affecting 27.5% of patients (95% CI: 14.9–45.1), with a high I^2^ value of 96%, as illustrated in Figure 9. The second most common respiratory sequela was shortness of breath, with a pooled prevalence of 12.0% (95% CI: 5.0–25.0) and an I^2^ value of 99%, as shown in Figure 10. Cough was also identified as a respiratory sequela associated with long COVID-19, with a pooled prevalence of 10.8% (95% CI: 4.9–22.3), as depicted in Figure 11.

### Cardiovascular sequelae of long COVID-19

**Figure 12:**
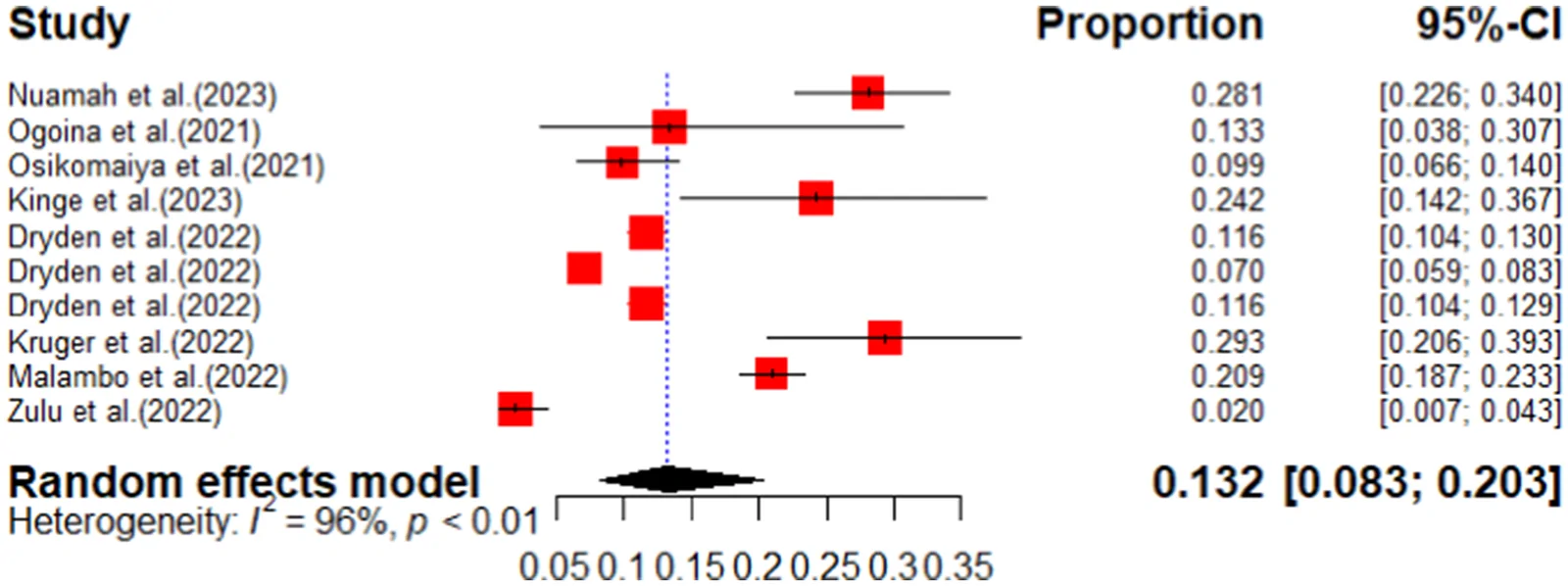
The forest plot chest pain health sequelae of long COVID-19 in sub-Saharan Africa.

**Figure 13:**
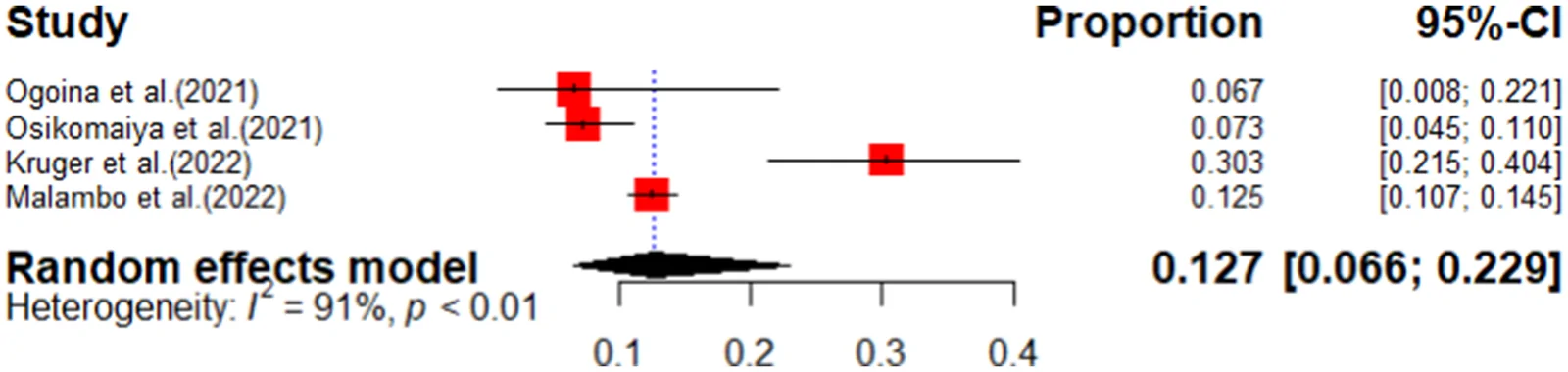
Forest plot of the pooled prevalence of palpitation as cardiovascular sequelae of COVID-19 in sub-Saharan Africa.

This systematic review and meta-analysis identified two primary cardiovascular sequelae associated with COVID-19. The first symptom, chest pain, was assessed across ten primary studies, revealing a pooled prevalence of 13.2% (95% CI: 8.3–20.3). This finding exhibited a high level of heterogeneity (*I*^2^ = 96.0%, *p*-value <0.01), as illustrated in Figure 12. The second long-term sequela examined was palpitations, which showed a pooled prevalence of 12.7% (95% CI: 6.6–22.4), also demonstrating significant heterogeneity (*I*^2^ = 91%, *p* < 0.01), as depicted in Figure 13. These results underscore the prevalence of these cardiovascular symptoms among individuals experiencing the long-term effects of COVID-19.

### General sequelae of long COVID-19

In this category, we have synthesized the combined prevalence of various symptoms associated with long COVID-19 in sub-Saharan Africa. Among these symptoms, fatigue exhibited the highest pooled prevalence at 17.4% (95% CI: 7.1–36.7), accompanied by a significant level of heterogeneity (I^2^) at 99.0%. The second most common symptom identified in this systematic review and meta-analysis was joint pain, with a pooled prevalence of 9.5% (95% CI: 7.3–12.3). Additionally, the pooled prevalence of fever as a long COVID-19 sequela was noted to be 3.1% (95% CI: 1.4–6.7), with a heterogeneity of 97%. A detailed overview of the general sequelae of long COVID-19 can be found in Table 5 below.

**Table 5:** The general sequelae of COVID-19 in sub-Saharan Africa.

COVID-19 sequelae	Studies	Proportion (95% CI)
Fatigue	Elias et al. (2023)	0.259 (0.213–0.309)
	Ogoina et al. (2021)	0.267 (0.123–0.459)
	Osikomaiya et al. (2021)	0.128 (0.091–0.173)
	Jassat et al. (2023)	0.028 (0.022–0.035)
	Jassat et al. (2023)	0.014 (0.010–0.019)
	Jassat et al. (2023)	0.012 (0.008–0.017)
	Kinge et al. (2023)	0.419 (0.295–0.552)
	Dryden et al. (2022)	0.509 (0.489–0.529)
	Dryden et al. (2022)	0.503 (0.480–0.526)
	Dryden et al. (2022)	0.508 (0.488–0.528)
	Kruger et al. (2022)	0.737 (0.639–0.821)
	Malambo et al. (2022)	0.385 (0.358–0.413)
	Zulu et al. (2022)	0.013 (0.004–0.034)
Random effect: *I*^2^ = 99%, *p* = 0.01		**0.174 (0.071–0.367)**
Joint pain	Elias et al. (2023)	0.132 (0.098–0.173)
	Ogoina et al. (2021)	0.133 (0.038–0.307)
	Osikomaiya et al. (2021)	0.088 (0.057–0.128)
	Kinge et al. (2023)	0.194 (0.104–0.314)
	Dryden et al. (2022)	0.060 (0.051–0.070)
	Dryden et al. (2022)	0.093 (0.081–0.108)
	Dryden et al. (2022)	0.060 (0.051–0.070)
	Malambo et al. (2022)	0.119 (0.101–0.138)
Random effect: *I*^2^ = 91%, *p* < 0.01		**0.095 (0.073–0.123)**
Fever	Nuamah et al. (2023)	0.206 (0.157–0.261)
	Ogoina et al. (2021)	0.033 (0.001–0.172)
	Osikomaiya et al. (2021)	0.062 (0.037–0.097)
	Dryden et al. (2022)	0.018 (0.13–0.024)
	Dryden et al. (2022)	0.015 (0.010–0.022)
	Dryden et al. (2022)	0.018 (0.13–0.024)
	Zulu et al. (2022)	0.013 (0.004–0.034)
Random effect: *I*^2^ = 97%, *p* < 0.01		**0.031 (0.014–0.067)**
Sore throat	Nuamah et al. (2023)	0.209 (0.161–0.265)
	Ogoina et al. (2021)	0.033 (0.001–0.172)
	Osikomaiya et al. (2021)	0.040 (0.020–0.071)
	Zulu et al. (2022)	0.007 (0.001–0.024)
Random effect: *I*^2^ = 99%, *p* < 0.01		**0.039 (0.009–0.145)**
Myalgia	Ogoina et al. (2021)	0.067 (0.008–0.221)
	Osikomaiya et al. (2021)	0.088 (0.057–0.128)
	Jassat et al. (2023)	0.009 (0.006–0.014)
	Jassat et al. (2023)	0.007 (0.004–0.010)
	Jassat et al. (2023)	0.003 (0.002–0.006)
	Kinge et al. (2023)	0.210 (0.117–0.33)
	Kruger et al. (2022)	0.0.485 (0.383–0.587)
	Malambo et al. (2022)	0.141 (0.122–0.162)
	Zulu et al. (2022)	0.007 (0.001–0.024)
Random effect: *I*^2^ = 99%, *p* < 0.01		**0.039 (0.011–0.123)**
Loss of appetites	Elias et al. (2023)	0.053 (0.032–0.082)
	Osikomaiya et al. (2021)	0.088 (0.057–0.128)
	Dryden et al. (2022)	0.005 (0.003–0.009)
	Dryden et al. (2022)	0.011 (0.007–0.016)
	Dryden et al. (2022)	0.005 (0.003–0.009)
	Zulu et al. (2022)	0.013 (0.004–0.034)
Random effect: *I*^2^ = 96%, *p* < 0.01		**0.016 (0.006–0.039)**

The bold values shows the “pooled result of the each sequalae.”

### Other system long sequalae of COVID-19

One of the musculoskeletal sequelae associated with COVID-19 is joint pain. A meta-analysis was conducted, pooling data from eight studies across four countries, which revealed a pooled prevalence of joint pain among COVID-19 patients at 9.5% (95% CI: 7.3–12.3%). In this systematic review, we also examined symptoms related to the ear, nose, and throat (ENT). Four primary studies were included in the analysis of sore throat. The pooled results indicated that the prevalence of sore throat among patients experiencing long COVID-19 is 3.9% (as shown in Table 6).

**Table 6:** Other system sequelae of COVID-19 in sub-Saharan Africa.

COVID-19 sequelae	Studies	Proportion (95% CI)
Joint pain	Elias et al. (2023)	0.132 (0.098–0.173)
	Ogoina et al. (2021)	0.133 (0.038–0.307)
	Osikomaiya et al. (2021)	0.088 (0.057–0.128)
	Kinge et al. (2023)	0.194 (0.104–0.314)
	Dryden et al. (2022)	0.060 (0.051–0.070)
	Dryden et al. (2022)	0.093 (0.081–0.108)
	Dryden et al. (2022)	0.060 (0.051–0.070)
	Malambo et al. (2022)	0.119 (0.101–0.138)
Random effect: *I*^2^ = 91%, *p* < 0.01		**0.095 (0.073–0.123)**
Fever	Nuamah et al. (2023)	0.206 (0.157–0.261)
	Ogoina et al. (2021)	0.033 (0.001–0.172)
	Osikomaiya et al. (2021)	0.062 (0.037–0.097)
	Dryden et al. (2022)	0.018 (0.13–0.024)
	Dryden et al. (2022)	0.015 (0.010–0.022)
	Dryden et al. (2022)	0.018 (0.13–0.024)
	Zulu et al. (2022)	0.013 (0.004–0.034)
Random effect: *I*^2^ = 97%, *p* < 0.01		**0.031 (0.014–0.067)**
Sore throat	Nuamah et al. (2023)	0.209 (0.161–0.265)
	Ogoina et al. (2021)	0.033 (0.001–0.172)
	Osikomaiya et al. (2021)	0.040 (0.020–0.071)
	Zulu et al. (2022)	0.007 (0.001–0.024)
Random effect: *I*^2^ = 99%, *p* < 0.01		**0.039 (0.009–0.145)**

### Meta-analysis of the factors associated with long COVID-19

The findings revealed that only comorbidity (diabetes and hypertension) was significantly associated with long COVID-19. The statistical analysis results revealed that older age and prolonged hospitalization did not show a significant association with long COVID-19 complications in this review. The pooled results showed that long hospitalization and advanced age did not demonstrate a statistically significant relationship with the long COVID-19 sequelae. The only factor found to be statistically significant associated with long COVID-19 sequelae was the presence of comorbidities (diabetes and hypertension) among the study participants. Individuals with comorbidities were found to be 4 times more likely (pooled OR = 4.34, 95% CI: 1.28–14.72) to experience long COVID-19 sequelae compared to those without comorbidities (Table 7).

**Table 7:** Factors associated with long COVID-19 sequelae in sub-Saharan Africa.

Factors	Number of studies included	Pooled OR (95% CI)	Heterogeneity, *p*-value
Comorbidity	2 [26, 27]	4.34 (1.28–14.72)	*I*^2^ = 86%, *p* < 0.01
Long hospitalization	2 [26, 27]	3.59 (0.31–41.83)	*I*^2^ = 97%, *p* < 0.01
Older age	3 [26, 28, 29]	0.84 (0.40–1.78)	*I*^2^ = 77%, *p* = 0.01

## Discussion

To our knowledge, this is the first meta-analysis exploring the prevalence, risk factors, and persistent symptomatology of COVID-19 in sub-Saharan Africa. Sixteen published studies were included, 3,656 papers were initially screened, and 16 published papers were included for analysis with a total sample size of 18,104 patients. The pooled prevalence of long COVID-19 sequelae in Sub-Saharan Africa was 42.1%. The most common long COVID-19 sequelae symptoms were pulmonary sequelae (27.5%), sleep disturbance (22.5%), brain fog (27.4%), fatigue (17.4%), and anxiety (22.3%). According to our systematic review and meta-analysis conducted in sub-Saharan Africa, the pooled incidence of long COVID-19 sequelae was found to be 42.1% (95% CI: 29.9–55.4). This finding is comparable to a previous study conducted in Africa, which reported a prevalence of long COVID-19 at 56.9% (26). Additionally, another study involving participants from Africa showed a pooled prevalence of 48.6% (27). However, the incidence found in the sub-Saharan Africa study was higher than the prevalence reported by Leon et al. (2022) among children and adolescents, which was 25.0% (28). The discrepancy in findings could potentially be attributed to variations in the study population and the countries where the studies were conducted.

According to our systematic review, the symptoms that can occur as a result of long COVID-19 include neurological, respiratory, psychiatric, cardiovascular, and constitutional issues. Among the neurological symptoms, the highest prevalence was observed in brain fog (27.4%), followed by loss of smell (7.5%), loss of taste (7.5%), blurring of vision (7.3%), and headache (6.1%). The lowest prevalence of neurological symptoms was found to be a lack of concentration (5.4%). These symptoms were more common and persistent in the long term (6 months or more after infection) compared to the mid-term assessment (3–6 months). This indicates that post-COVID-19 syndrome is a significant long-term global public health concern that affects both hospitalized and non-hospitalized patients. This finding is comparable with a systematic review conducted by Natarajan et al. (29), Brain fog, concentration problems, and headaches were also observed as long symptomatologies in a meta-analysis conducted in 2022 (30).

The subgroup analysis revealed that South Africa exhibited the highest prevalence of COVID-19 sequelae, with a rate of 59.0% (95% CI: 44.9–71.7). This finding aligns with previous research conducted across the African continent. Several factors may contribute to this elevated prevalence (27). First, South Africa has faced a significant burden from COVID-19, which may have heightened the visibility and recognition of sequelae associated with the virus. The methodologies employed for assessing these sequelae in the country are likely to be both accurate and well-defined, enhancing the reliability of the findings. Moreover, the quality of healthcare services and the effectiveness of testing strategies for COVID-19 in South Africa play a crucial role in this context (31–33). The country’s healthcare infrastructure may facilitate more comprehensive evaluations of COVID-19 sequelae, allowing for more accurate reporting and understanding of the long-term impacts of the virus. Additionally, numerous studies conducted by scholars in South Africa regarding COVID-19 sequelae may further contribute to the observed high prevalence. These studies not only enrich the existing body of knowledge but also underscore the importance of ongoing research in this area. In summary, the combination of a high COVID-19 burden, effective assessment strategies, quality healthcare services, and a robust research environment collectively contribute to the notable prevalence of COVID-19 sequelae in South Africa.

This systematic review and meta-analysis also identified the psychiatric symptomatology. Of the psychiatric sequelae, the highest long COVID-19 symptom was sleep disturbance (22.5%), followed by anxiety (22.3%). This psychiatric symptomatology was also observed in the previous studies conducted among children and adolescents (28), the study conducted by Iqbal et al. (34), and the other systematic review conducted by Premraj et al. (30) which showed that sleep disturbance and anxiety were the identified psychiatric sequelae for long COVID-19. The establishment of clear diagnostic criteria for neurological and neuropsychiatric post-COVID-19 syndrome, along with a standardized approach to the multidisciplinary follow-up of COVID-19 patients, could play a crucial role in reducing the impact of the disease (35). Some authors speculate that SARS-CoV-2 could potentially trigger neurodegenerative diseases such as Multiple sclerosis, Parkinson’s disease, and narcolepsy in individuals who are already predisposed to these conditions. There have been reports of patients experiencing loss of smell and loss of taste, which may be associated with early signs of Parkinson’s disease (36–38). Additionally, there has been a significant increase in newly diagnosed psychiatric disorders such as anxiety, depression, insomnia, and dementia compared to unaffected individuals. Studies have shown that a considerable impact on mental health should be expected after recovering from COVID-19, similar to the prevalence of depression, anxiety, and posttraumatic stress disorder observed in previous outbreaks (39). Additionally, it is important to raise awareness about the occurrence of neurological and neuropsychiatric post-COVID-19 syndrome and conduct further research to explore interventions that can improve long-term outcomes, alleviate the burden of the disease, and enhance quality of life.

Other long-term sequelae of COVID-19 pooled in this systematic review were respiratory, which consists of cough, shortness of breath, and general pulmonary sequelae. The highest respiratory symptomatology was general pulmonary sequelae, with a pooled prevalence of 27.5%. The finding is comparable with the study conducted by Premraj et al. (27) and Iqbal et al. (28) which showed that COVID-19 had respiratory sequelae among COVID-19-confirmed cases. While most patients have fully recovered or are showing clinical improvement from COVID-19, some individuals may develop long-term pulmonary sequelae. As a result, certain patients may still experience cough and shortness of breath even 12 weeks after being hospitalized for COVID-19 (40). Additionally, research from the SARS pandemic suggests that lung damage may persist for up to 15 years in some patients (41). This implies radiological and critical intervention is also necessary for long-term complications of SARS-CoV-2.

Moreover, this systematic review examined the symptomatology of cardiovascular sequelae in Sub-Saharan Africa among individuals with long COVID-19. The primary cardiovascular symptoms identified were chest pain (13.2%) and palpitations (12.7%). These findings align with previous research conducted on a large sample of 47,910 patients (28) as well as with several systematic reviews conducted in different regions worldwide (42–44). It is worth noting that cardiovascular abnormalities were observed even in individuals with mild COVID-19 (45). This underscores the importance of assessing and intervening early in cardiometabolic disorders to alleviate the suffering of survivors after hospital discharge. The potential risks, such as myocarditis leading to sudden cardiac death, should not be overlooked, as individuals may still face the possibility of developing coronary artery disease, atrial fibrillation, or ventricular arrhythmias due to myocardial injury, despite apparent recovery of cardiac function. Taking proactive measures in managing these conditions is crucial to save the lives of survivors (46).

The constitutional symptoms were observed as long COVID-19 sequelae in sub-Saharan Africa. The highest pooled symptomatology of constitutional symptoms was fatigue (17.4%) followed by joint pain (9.5%). The other constitutional symptoms were myalgia (3.9%), loss of appetite (1.6%), and fever (3.1%). This symptomatology was also observed in different scholars in different regions of the world (47–50). Previous literature has reported comparable rates of fatigue following coronavirus epidemics. There is growing evidence suggesting that a significant number of individuals recovering from COVID-19 may experience persistent fatigue for weeks after the acute infection has resolved. Similar cases of postinfectious fatigue, occurring months or even years after recovery, have been observed in various viral infections. This long-term fatigue, which can meet the diagnostic criteria for chronic fatigue syndrome, appears to be more prevalent in individuals under 30 years old following epidemics of influenza A(H1N1) virus, SARS-CoV-2, Ebolavirus, and West Nile virus. The underlying cause of this chronic fatigue is believed to be a miscommunication in the inflammatory response pathways, particularly the cytokine networks. Additionally, there is a hypothesis that a COVID-19 infection may increase the susceptibility to various types of cancer. Hays et al. suggested that the activation of the MAPK and JAK–STAT pathway, along with a weakened immune system resulting from a cytokine storm, could potentially contribute to increased tumorigenesis following a COVID-19 infection (51–53).

The association between older age and long COVID-19 sequelae was conducted in this systematic review and meta-analysis. The association between older age and long COVID-19 sequelae was not statistically significant [POR = 0.84 (95% CI: 0.40, 1.78)], while older age was suggestively associated with long COVID-19 in previous studies (54–59). Recognizing the importance of post-COVID-19 conditions, it is worth noting that these conditions primarily impact individuals who have successfully overcome the acute phase of COVID-19 infection. Older individuals, especially those with multiple underlying comorbidities, are at a higher risk of severe illness and may not survive the acute phase of the virus. Post-COVID-19 conditions are specific to the population of COVID-19 survivors and are not indicative of the epidemiological characteristics of COVID-19 (60–62). One possible explanation for the lack of correlation between older age and long COVID-19 sequelae could be the younger population in sub-Saharan Africa, where age-based interventions for long COVID-19 may not be necessary. Therefore, it is essential to consider geographic location and demographic-based programs and interventions to improve the quality of life of successful COVID-19 survivors.

There is a significant association between comorbidity and long COVID-19 in this systematic review, with a pooled odds ratio of 4.34 (95% CI: 1.28–14.72). The finding is comparable with the findings of the previous studies (56, 57, 63, 64). The possible justification for this finding could be decreased immunity and increased deterioration of the patient with comorbidity. The presence of preexisting medical conditions, known as comorbidities, may contribute to the risk of developing long COVID-19. Among these comorbidities, obesity appears to be associated with an increased risk. However, it is important to approach this assumption with caution at this stage. This is because potential confounding factors, particularly those related to hospitalization, have not been adequately controlled for in the studies conducted so far. It is worth noting that obese patients tend to have more severe COVID-19 disease and higher hospitalization rates compared to non-obese patients. Furthermore, the association of long COVID-19 with other medical comorbidities, such as diabetes or organ transplants, has only been investigated in a single prior study. Therefore, more research studies are needed in the future to fully understand the relationship between comorbidities and the risk of developing long COVID-19 (65). This suggests that individuals with preexisting medical conditions may be more susceptible to experiencing prolonged effects following the COVID-19 infection. It is important to note that these findings are based on the analysis of existing studies and should be interpreted in the context of their limitations.

According to our systematic review and meta-analysis, there appears to be no significant association between long hospitalization and long COVID-19 sequelae. This finding is in contrast to previous literature that has suggested a link between prolonged hospital stays and the development of long COVID-19 (56, 59, 64, 66). In fact, a recent study has found that individuals with indications of fibrosis on CT chest imaging who suffer from long COVID-19 tend to have longer hospital stays and a higher frequency of intensive care admissions. Furthermore, this particular group of patients often requires an extended duration of pulsed steroid therapy and antiviral treatment. Additionally, this specific group of patients often requires an extended duration of pulsed steroid therapy and antiviral treatment (67, 68). It is important to note that the low incidence of long hospitalization and severity of COVID-19 in Africa may explain the lack of association between hospitalization and long COVID-19 in the aforementioned study. Nonetheless, it is crucial to continue researching and understanding the complex nature of COVID-19 and its potential risk factors to provide the best possible care and treatment for those affected.

### Strengths and limitations of the study

This systematic review and meta-analysis boasts several strengths. Notably, it incorporates data from multiple countries and adheres to established systematic review guidelines. Additionally, the pooled analysis provides robust evidence, further enhancing the study’s credibility. However, the study does have some limitations. One significant concern is that the assessment of long COVID-19 sequelae relied on self-reported data rather, which may risk recall bias. Furthermore, the study did not take into account the health status and availability of healthcare services within the communities. Finally, the review focused exclusively on published articles in English, which may limit its applicability, particularly in sub-Saharan Africa, where many countries speak French and Portuguese. This language barrier could result in different outcomes that were not captured in the analysis.

## Conclusion

In this systematic review and meta-analysis, we identified the neurological, psychiatric, respiratory, cardiovascular, and general symptoms associated with long COVID-19. Our findings indicated that in sub-Saharan Africa, 4 out of every 10 confirmed COVID-19 cases developed long COVID-19 sequelae. The most common symptoms included pulmonary sequelae, sleep disturbances, brain fog, fatigue, and anxiety. Individuals with comorbidities, such as diabetes and hypertension, were at increased risk for these sequelae.

To reduce the occurrence of long COVID-19 sequelae, multi-sectoral and multifaceted interventions that take into account the specific needs of populations with comorbidities are crucial. Additionally, close monitoring and follow-up care can further mitigate the risk of developing these long-term effects. Therefore, it is essential to design targeted interventions that are tailored to the geographic and demographic characteristics of affected populations.

## Data Availability

The original contributions presented in the study are included in the article/Supplementary material, further inquiries can be directed to the corresponding author/s.

## Supplementary material

^2^R was created in 1992 by Ross Ihaka and Robert Gentleman from the University of Auckland.
