# Correction: The prolonged health sequelae “of the COVID-19 pandemic” in sub-Saharan Africa: a systematic review and meta-analysis

**DOI:** 10.3389/fpubh.2026.1979047

**Published:** 2026-09-29

**Authors:** Melsew Setegn Alie, Getayeneh Antehunegn Tesema, Gossa Fetene Abebe, Desalegn Girma

**Affiliations:** 1 Department of Public Health, School of Public Health, College of Medicine and Health Science, Mizan-Tepi University, Mizan-Aman, Ethiopia; 2 Department of Epidemiology and Biostatistics, Institute of Public Health, College of Medicine and Health Sciences, University of Gondar, Gondar, Ethiopia; 3 School of Public Health and Preventive Medicine, Monash University, Melbourne, VIC, Australia; 4 Department of Midwifery, College of Medicine and Health Science, Mizan Tepi University, Mizan-Aman, Ethiopia

**Keywords:** long COVID-19, COVID sequalae, COVID-19, SARS CoV-2, sub-Saharan Africa

There was a mistake in Table 1 as published. Each included study was not cited as a reference. The corrected table citing the references appears below.

**Table 1:** Descriptive presentation of the study included in systematic review and meta-analysis.

References	Country	Sample size	Population	Study period
Elias et al. (1)	Ethiopia	340	General population	June 12, 2020 and November 1, 2021
Engida et al. (2)	Ethiopia	573	General population	June 1 to 20, 2020
Seyoum et al. (3)	Ethiopia	405	General population	January 2021 and January 2022
Nuamah et al. (4)	Gahanna	253	General population	April 1, 2020 to March 31, 2021
Ogoina et al. (5)	Nigeria	30	General population	April and December 2020
Osikomaiya et al. (6)	Nigeria	274	General population	April and June 2020
Jassat et al. (7)	South Africa	2,366	Hospitalized patients	Not reported
Jassat et al. (7)	South Africa	2,840	Hospitalized patients	Not reported
Jassat et al. (7)	South Africa	2,626	Hospitalized patients	Not reported
Kinge et al. (8)	South Africa	62	Health workers	15 February to 15 April 2021
Dryden et al. (9)	South Africa	2,410	General population	Dec 1, 2020 and August 23, 2021
Dryden et al. (9)	South Africa	1,873	General population	Dec 1, 2020 and August 23, 2021
Dryden et al. (9)	South Africa	2,413	General population	December 2020 and August 2021
Kruger et al. (10)	South Africa	99	Long COVID-19 patients	Not reported
Malambo et al. (11)	Zambia	1,238	General population	August 2020 to January 2023
Zulu et al. (12)	Zambia	302	General population	March 18th, 2020 to March 30th, 2021

The original version of this article has been updated.
