# Supplementary material for: The prolonged health sequelae “of the COVID-19 pandemic” in sub-Saharan Africa: a systematic review and meta-analysis
Source: Front Public Health. 2025 Jan 24;13:1415427. doi: 10.3389/fpubh.2025.1415427 (PMC11803863; doi:10.3389/fpubh.2025.1415427)
Supplement: Supplementary file 1 [file Table_1.docx]

| Authors(year) | Country | Sample size | Population | Study period | NOS |
| --- | --- | --- | --- | --- | --- |
| Elias et al.(2023) | Ethiopia | 340 | General population | June 12, 2020, and November 1, 2021 | 7 |
| Engida et al.(2021) | Ethiopia | 573 | General population | June 1 to 20, 2020 | 8 |
| Seyoum et, al.(2023) | Ethiopia | 405 | General population | January 2021 and January 2022 | 6 |
| Nuamah et al.(2023) | Gahana | 253 | General population | Aprile 1, 2020 to March 31,2021 | 7 |
| Ogoina et al.(2021) | Nigeria | 30 | General population | April and december 2020 | 8 |
| Osikomaiya et al.(2021) | Nigeria | 274 | General population | April and June 2020 | 8 |
| Jassat et al.(2023) | South Africa | 2366 | hospitalized patient | Not reported | 7 |
| Jassat et al.(2023) | South Africa | 2840 | hospitalized patient | Not reported | 6 |
| Jassat et al.(2023) | South Africa | 2626 | hospitalized patient | Not reported | 8 |
| Kinge et al.(2023) | South Africa | 62 | health worker | 15 February to 15 April 2021 | 6 |
| Dryden et al.(2022) | South Africa | 2410 | General population | Dec 1, 2020, and Aug 23, 2021 | 8 |
| Dryden et al.(2022) | South Africa | 1873 | General population | Dec 1, 2020, and Aug 23, 2021 | 8 |
| Dryden et al.(2022) | South Africa | 2413 | General population | December 2020 and August 2021 | 7 |
| Kruger et al.(2022) | South Africa | 99 | Long COVID-19 patients | Not reported | 7 |
| Malambo et al.(2022) | Zambia | 1238 | General population | Aug-2020 to Jan-2023 | 8 |
| Zulu et al.(2022) | Zambia | 302 | General population | March 18th, 2020 to March 30th, 2021 | 8 |

NOS= Newcastle-Ottawa Scale
