# Supplementary figures and images for: The prolonged health sequelae “of the COVID-19 pandemic” in sub-Saharan Africa: a systematic review and meta-analysis
Source: Front Public Health. 2025 Jan 24;13:1415427. doi: 10.3389/fpubh.2025.1415427 (PMC11803863; doi:10.3389/fpubh.2025.1415427)

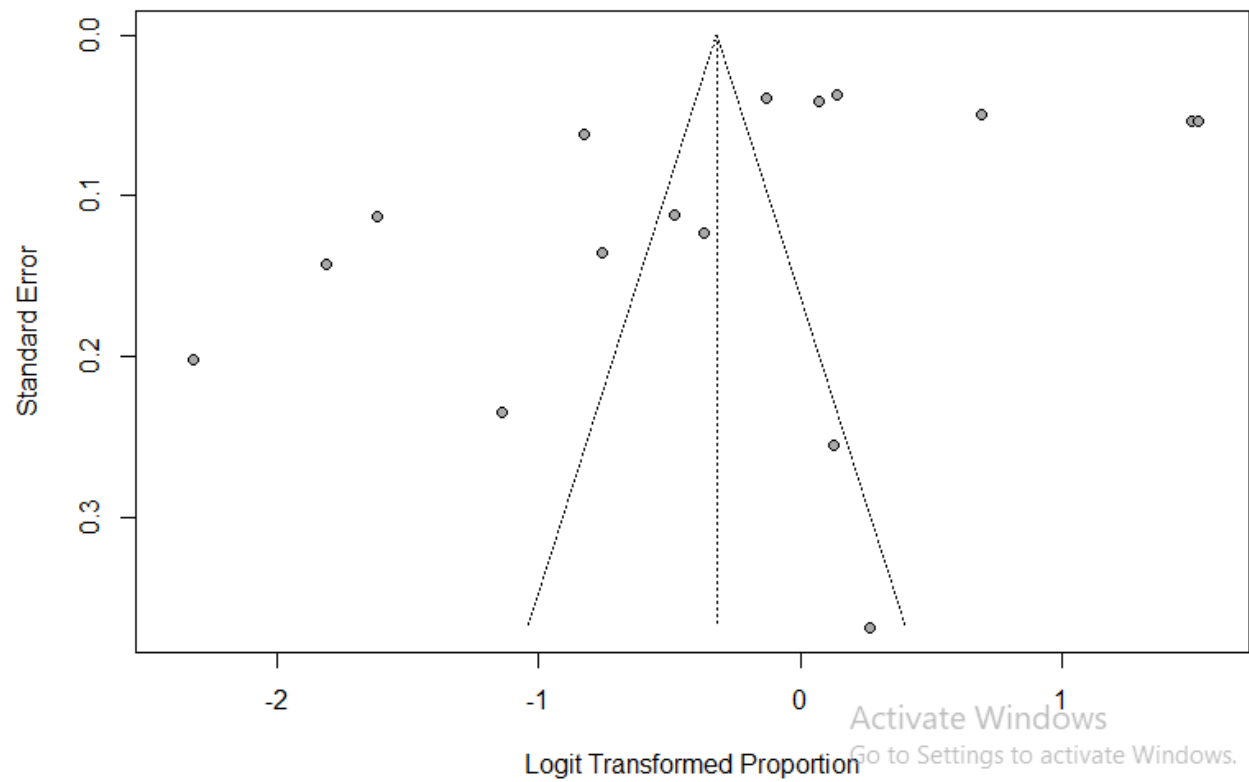

Supplementary figure 1: Show the funnel plot of the study on COVID-19 sequelae in Sub-Saharan Africa

Supplement: Supplementary file 2 [file Image_1.pdf]
